# On mathematical modelling of measles disease via collocation approach

**DOI:** 10.3934/publichealth.2024032

**Published:** 2024-05-06

**Authors:** Shahid Ahmed, Shah Jahan, Kamal Shah, Thabet Abdeljawad

**Affiliations:** 1 Department of Mathematics, Central University of Haryana, Mohindergarh-123031, India; 2 Department of Mathematics and Sciences, Prince Sultan University, Riyadh 11586, Saudi Arabia

**Keywords:** fractional SEIR modeling, fixed point theory, Haar wavelet, numerical analysis, Ulam-Hyers stability

## Abstract

Measles, a highly contagious viral disease, spreads primarily through respiratory droplets and can result in severe complications, often proving fatal, especially in children. In this article, we propose an algorithm to solve a system of fractional nonlinear equations that model the measles disease. We employ a fractional approach by using the Caputo operator and validate the model's by applying the Schauder and Banach fixed-point theory. The fractional derivatives, which constitute an essential part of the model can be treated precisely by using the Broyden and Haar wavelet collocation methods (HWCM). Furthermore, we evaluate the system's stability by implementing the Ulam-Hyers approach. The model takes into account multiple factors that influence virus transmission, and the HWCM offers an effective and precise solution for understanding insights into transmission dynamics through the use of fractional derivatives. We present the graphical results, which offer a comprehensive and invaluable perspective on how various parameters and fractional orders influence the behaviours of these compartments within the model. The study emphasizes the importance of modern techniques in understanding measles outbreaks, suggesting the methodology's applicability to various mathematical models. Simulations conducted by using MATLAB R2022a software demonstrate practical implementation, with the potential for extension to higher degrees with minor modifications. The simulation's findings clearly show the efficiency of the proposed approach and its application to further extend the field of mathematical modelling for infectious illnesses.

## 1. Introduction

Mathematical epidemiology is the use of mathematical models and methodologies to gain insights into the transmission and behavior of contagious diseases among populations. This discipline holds a crucial place in epidemiological investigations and contributes to the formulation of public health strategies. These mathematical models simulate disease transmission, predict outbreaks, assess the impact of interventions (such as vaccination campaigns or social distancing measures), and evaluate the effectiveness of various control strategies. We refer to [1]–[4]. These models incorporate variables such as population size, disease transmission rates, incubation periods, and other epidemiological parameters, giving important information on how infectious illnesses behave and directing public health initiatives. Mathematical modeling has proven to be valuable in the investigation and control of diseases like COVID-19, TB virus, influenza and Ebola disease, waterborne disease, hepatitis B virus and rabies virus, etc. For the mentioned diseases which have been studied by using mathematical models, we refer to [5]–[7].

Fractional calculus (FC) distinguishes itself from classical differential and integral calculus in how it applies integral and differential operators. Fractional order differential equations (FODEs) have been crucial in efforts to improve our understanding of many diseases, resulting in the development and investigation of mathematical models for a variety of diseases [8]. The use of FC has increased its value as a useful research tool in a number of fields across various domains in the realms of basic sciences and engineering. One key feature of fractional derivatives is their ability to manage integrals and derivatives of any order, regardless of whether they are real or complex. This unique property gives them a nonlocal quality, meaning that the future condition depends not just on the present state but also on all past states. Within the framework of FC, three distinct types of fractional differential operators exist. The Riemann-Liouville (RL) and Caputo relies on the power-law kernel [9]. The Caputo-Fabrizio derivative is rooted in decay processes [10], [11]. Finally, the Mittag-Leffler law encompasses both the power law and exponential decay. These properties can be effectively described by using Atangana–Baleanu fractional-order derivatives. These remarkable characteristics have been utilized in various fields, encompassing mathematics, applied sciences, engineering, biology and physics [12]. Authors [13] derived and simulated the numerical solutions for several control techniques in different fractional orders by using the iterative fractional-order Adams-Bashforth methodology. Researchers have made significant attempts to investigate this specific topic, exploring it from many perspectives which include both theoretical and numerical investigations. They developed an extensive number of methodologies and processes, each specifically to provide theoretical, analytical, and numerical results such as unraveling pine wilt disease by using a spectral method [14], the transmission dynamics and sensitivity analysis of pine wilt disease through the use of a fractal fractional operator [15], the sensitivity analysis of COVID-19 [16], the development of a fractional order model with non-local kernels [17], the fractional mathematical modeling of malaria disease and typhoid fever disease [18], [19], analysis and optimal control of SEIRI epidmic model [20]. Also, the numerical solution of the nonlinear delay integrodifferential equation using a wavelet [21], the variable-order fractional differential equation using Haar wavelet [22], Ulam- type stability of the impulsive delay integrodifferential equation [23] the computational modeling of a measles epidemic in human population [24], the co-dynamics of measles and dysentery disease [25], and the modeling of childhood disease outbreak in a community with the inflow of susceptible and vaccinated new-born [26]. These approaches have proven to be quite useful in the study of a wide range of problems, including difficulties relating to existence, approximation, and stability theory, among others [27]. Solving fractional differential equations (FDEs) to obtain accurate or analytical solutions can be time-consuming. In response to this difficulty, numerous tools and methodologies have arisen in recent decades to handle this topic and achieve approximations or analytical results. Techniques such as the decomposition method [28], transform method, and perturbation method [29] are examples of analytical and semi-analytical procedures. Numerical approaches have been created to identify approximate solutions to various FDEs problems. Spectral methods [30] are examples of notable numerical techniques.

Wavelet analysis has become a popular topic of research in several scientific and technical domains. Wavelets are viewed as a fresh basis for functions by a number of scholars and as a tool for time-frequency analysis by others. Given that wavelets are a flexible tool with many mathematical components and several potential uses, it is obvious that all of these are accurate [31], [32]. By using wavelet techniques, we can break down a complicated function into several smaller ones and study each one separately at various scales. This feature, along with a fast wavelet approach, makes these methods very interesting for analysis and synthesis. Wavelet-based collocation techniques have become more popular in numerical analysis because of their fast convergence, low computational cost, and straightforward procedure [33]. Wavelet techniques are a relatively recent addition to the family of orthogonal functions, with notable and attractive properties such as orthogonality, compact support, unconstrained regularity and good localization. As a result, they are frequently used to derive the numerical solutions to several mathematical models that are being developed in the fields of biology, chemistry, and physical sciences. Various types of wavelets, such as Chebeshev [34], the Haar method [35], etc. Hermite technique were used by researchers [36] to compute numerical solutions to infectious disease model. Authors [37] applied Bernstein wavelets to study a biological model. These wavelet-based approaches not only possess a strong mathematical foundation that also exhibit the capability to tackle nonlinear problems effectively. Among the various types of wavelets, Haar wavelets hold a distinct place. They are characterized by a pairwise constant function that forms the Haar wavelet basis series, making them one of the simplest wavelet series in mathematics. The Haar wavelet's orthogonality, local support, and simplicity make it an effective choice for use in the derivation of the numerical solution of FODEs. Recently, the Haar wavelets approach has been used to solve the HIV infection fractional model [38], the SEIR epidemic model [39]. The ability of Haar wavelets to efficiently capture complex nonlinear dynamics while maintaining high accuracy makes them particularly well-suited for handling the intricacies of fractional-order models. Moreover, the robustness of Haar wavelet methods when dealing with nonlinearities, coupled with their computational speed, significantly contributes to efforts to model the dynamic nature of measles disease. Given the inherently nonlinear nature of epidemiological systems and the fractional-order dynamics involved in measles transmission, researchers may improve the efficacy of measles dynamics simulations and analyses by making use of Haar wavelet approaches, which not only minimize computer cost they also increase model correctness and dependability. Due to the advantages offered by Haar wavelets when applied to solve the nonlinear models of fractional order, we are motivated to use the Haar wavelet collocation method (HWCM) to address the nonlinear fractional model of measles disease. The aim was to analyze the dynamics of the system with the utmost precision and minimal error. This work's major goal was to present and explore an HWCM that uses the Haar wavelet basis to understand the numerical and geometrical behavior of a nonlinear mathematical model. The findings in this research are original and have not previously been reported in the literature. This technique eliminates complex numerical methods and yields valuable insights into the numerical behavior of the model. The proposed solution also uses numerical computing to tackle the problem. Researchers have also used other numerical and analytical techniques to study various biological models, we refer to [40]–[42]. Also researchers have deduced various results devoted to mathematical analysis including the existence theory, and Ulam-Hyers stability criteria which have now a days extended to mathematical problems of physical importance. For mentioned results, we refer to [43]–[46].

### 1.1. Structure of the model

**Figure 1.**
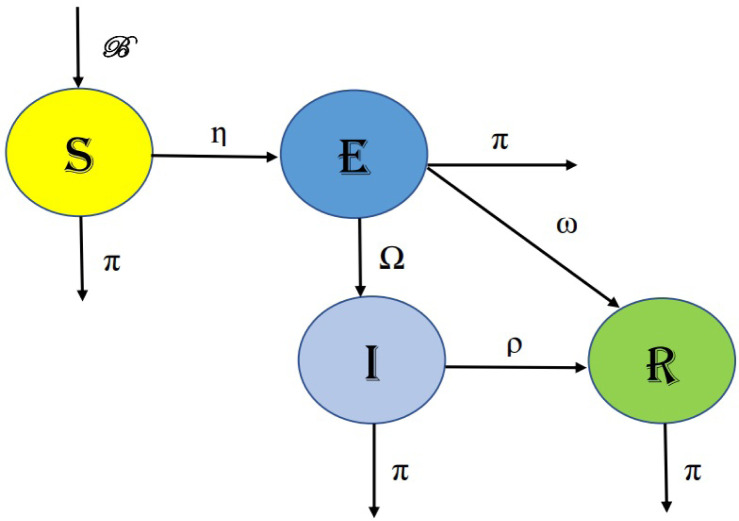
Flow chart.

In several infectious diseases, there is an initial latent period following exposure corresponding to the period before individuals become infectious. This latent period is a crucial aspect of disease progression and cannot be overlooked when analyzing infectious stages. Consequently, it is sensible to incorporate an initial compartment into epidemiological models. We have created a clear and predictable mathematical model to explain the process of measles transmission. In order to construct the model, the entire population (N) is segmented into four distinct categories: susceptible $(\mathbb{S})$, exposed $(\mathbb{E})$, infected $(\mathbb{I})$, and recovered $(\mathbb{R})$. In Figure 1, we have detailed the changes that occurred between these groups. The susceptible class, denoted as $(\mathbb{S})$, experiences an increase due to births or immigration at a rate represented by $\mathcal{B}$. At a rate *π*, natural death also has an impact. At a rate *η*, infection results from contact with infected people. At a rate *η*, interaction with infected individuals results in the generation of a class $\mathbb{E}$, which is the group of exposed individuals. This class decreases as a results of testing and treatment for measles at a rate of *ω*, transitioning to the infected class at a rate of Ω. In addition to these factors, the population in this class is also influenced by the natural mortality rate denoted as *π*. The group of infected people, denoted as class $\mathbb{I}$, emerges as a result of the transition of exposed peoples at a rate of Ω. It is decreased at a rate of *ρ* through infection recovery and at a rate of *π* due to natural mortality. The model requires the assumption that both recovered exposed persons and recovered infected persons develop a lifelong immunity to the illness. As a result, a class $\mathbb{R}$ is created, which is made up of those who have total immunity to the illness. Natural mortality affects this class of recovered people at a rate of *π* [40], [41].

**Table 1.** Parameters of the fractional SEIR model.

Parameter	Description
$\mathcal{B}$	Birth rate
*ρ*	Rate of recovery from infection
*η*	Rate of infected individuals
*π*	Natural death rate
Ω	Infected rate
*ω*	Measles therapy rate

Consequently, the deterministic model's diagram is as shown in Figure 1.

The following forms are used to present the fractional order SEIR model [27], [40], [42].

1.1 \begin{equation*} 	\begin{aligned} 		\dfrac{d\mathbb{S}(\tau)}{d\tau} &=\mathcal{B}-\eta\mathbb{S}(\tau)\mathbb{I}(\tau)-\pi\mathbb{S}(\tau), \\ 		\dfrac{d\mathbb{E}(\tau)}{d\tau} &=\eta\mathbb{S}(\tau)\mathbb{I}(\tau)-(\omega+\pi+\Omega)\mathbb{I}(\tau), \\ 		\dfrac{d\mathbb{I}(\tau)}{d\tau} &= \Omega\mathbb{E}(\tau)-(\rho+\pi)\mathbb{I}(\tau), \\ 		\dfrac{d\mathbb{R}(\tau)}{d\tau} &= \rho\mathbb{I}(\tau)+\omega\mathbb{E}(\tau)-\pi\mathbb{R}(\tau). 	\end{aligned} \end{equation*}

$N(\tau) = \mathbb{S}(\tau) + \mathbb{E}(\tau) + \mathbb{I}(\tau) + \mathbb{R}(\tau), \forall \tau$. According to (1.1), $(\mathbb{S} + \mathbb{E} + \mathbb{I} + \mathbb{R})^\prime = 0$; thus, $N(\tau)$ is constant and equal to *N*. System (1.1) is in a feasible region because $\Delta = \{(\mathbb{S} + \mathbb{E} + \mathbb{I} + \mathbb{R}) : 0 \leq \mathbb{S}, \mathbb{E}, \mathbb{I}, \mathbb{R} \leq N\}$. All associated parameters and state variables in the model stay non-negative while it follows the human population, where $\tau\geq 0$.

The remaining sections of this article are structured as follows. Section 2 discusses the preliminaries and fractional model formulation. Section 3 elaborates on the theoretical properties associated with the fractional model and qualitative analysis. In Section 4, we establish essential conditions for the Ulam-Hyers stability (UHS) of the solution within the context of the model under consideration. Section 5 presents the numerical scheme, along with graphical results and a corresponding discussion. Lastly, in Section 6, the conclusion is drawn.

## 2. Preliminaries

Fractional-order models have garnered a significant amount of attention across various scientific disciplines and have been the focus of extensive research. Our exploration begins with the concepts of fractional-order integration and differentiation, as described in [9], [10]. We provide a brief overview of key lemmas and definitions from FC that are essential for studying the proposed model.

**Definition 2.1.** *[1] For any function $\Theta \in L^1([0, \infty), \mathbb{R})$ the RL integral with order $\chi \in (0, 1)$ is given by*

$$I_{0^{+}}^{\chi }\Theta (\tau )=\frac{1}{\Gamma (\chi )}\int_{0}^{\tau }{\left(\tau -s\right)}^{\chi -1}\Theta (s)ds.$$

**Definition 2.2.** *[1] For any function Θ the fractional order $\chi$ Caputo derivatives is defined as follows:*

$$D_{0^{+}}^{\chi }\Theta (\tau )=\frac{1}{\Gamma (n-\chi )}\int_{0}^{\tau }{\left(\tau -s\right)}^{n-\chi -1}\Theta^{n}(s)ds.$$

**Definition 2.3.** *The subsequent equation holds true:*

$ 	I^a\left[^{c}D^\chi\Theta\right](\tau) = \Theta(\tau) + b_0 + b_1\tau + b_2\tau^2 + \ldots + b_{n-1}\tau^{n-1}, 	$

where r=0,1,…,n−1, with $b_r \in \mathbb{R}$, and $n = \lfloor \Theta \rfloor + 1$.

**Lemma 2.4.** *Given g as a compact and continuous mapping from the Banach space $\mathcal{B}$ → $\mathcal{D}$, where $\mathcal{B}$ is characterized by elements $\mathcal{X} \in \mathcal{B}$ such that $\mathcal{X} = \Upsilon g \mathcal{X}$ with $\Upsilon$ in the interval* [0, 1]*, when $\mathcal{D}$ is bounded, it implies that for the function g there exists at least one fixed point*.

### 2.1. Haar wavelet

Haar wavelet function $\mathcal{H}(\tau)$, along with its corresponding Haar scaling function denoted by $\widetilde{\mathcal{H}}_0(\tau)$ defined as:

$ \mathcal{H}(\tau) = \begin{cases} 	1, &  \tau \in \left[{0}, \frac{1}{2}\right), \\ 	-1, &  \tau \in \left[\frac{1}{2}, 1\right), \\ 	0, & \text{otherwise}, \end{cases} $

$ \text{if} \quad \tau \in [0, 1),\quad \widetilde{\mathcal{H}}_0(\tau) = 1. $

On [0, 1), multi-resolution analysis produces a range of Haar wavelets, each of which can be represented as $\widetilde{\mathcal{H}}_m(\tau)$ [34]. As a result, this leads to the subsequent relationship:

$ \widetilde{\mathcal{H}}_s(t) = 2^{j/2}\mathcal{H}\left(2^j\tau - p\right),\quad s = 1, 2, \ldots , $

where $s{=2}^{i}+q$, $q=0,1,\ldots {,2}^{i}-1$, and i=0,1,…. Furthermore, we perform a translation of the Haar functions on the interval v−1≤τ<v, as follows

$ \widetilde{\mathcal{H}}_{v,s}(\tau) = \widetilde{\mathcal{H}}_s(\tau + 1 - v),\quad \text{for } \ v = 1, 2, \ldots,\ \rho,\quad s = 0, 1, 2, \ldots,\ \text{where } \rho \in \mathbb{N}. $

From [34] we conclude that the sequence $ \left\langle \widetilde{\mathcal{H}}_s(\tau) \right\rangle_{s=0}^\infty $ constitutes a comprehensive orthonormal system within the space $L^{2}[0,1)$. Meanwhile, the sequence

$ \left\langle \widetilde{\mathcal{H}}_{v,s}(\tau) \right\rangle_{s=0}^\infty,\ v = 1, 2, \ldots, \rho, $

is orthonormal in $L^{2}[0,\rho )$. This signifies that any function g(τ) within the domain $L^{2}[0,\rho )$ could represent the Haar orthonormal basis functions in series, as follows

$  g(\tau) = \sum_{v=1}^\rho \sum_{s=0}^\infty C_{v,s} \widetilde{\mathcal{H}}_{v,s}(\tau).$

Moreover, when this series is truncated, we obtain an approximate equivalent, denoted as $y_{q}(\tau )$, for g(τ):

$ g(\tau) \approx y_q(\tau) = \sum_{v=1}^\rho \sum_{s=0}^{q-1} c_{v,s} \widetilde{\mathcal{H}}_{v,s}(\tau) = B^T_{\rho q \times 1} \widetilde{\mathcal{H}}_{\rho q \times 1}(\tau),$

Here, the coefficients denoted by $C_{v,s}$ can be calculated through the use of the inner product as for s=1,2,…,(q−1), v=1,2,…,ρ,

$ \langle g(\tau), \widetilde{\mathcal{H}}_{v,s}(\tau) \rangle = \int_{v-1}^v g(\tau) \widetilde{\mathcal{H}}_{v,s}(\tau) \, d\tau, $

$ B^T_{\rho q \times 1} = \begin{bmatrix} 	C_{1,0}, & \ldots, & C_{1,p-1}, & C_{2,0}, & \ldots, & C_{2,p-1}, & \ldots, & C_{\rho,0}, & \ldots, & C_{\rho,q-1} \end{bmatrix} $

$ \widetilde{\mathcal{H}}_{\rho q \times 1}^T = \begin{bmatrix} 	\widetilde{\mathcal{H}}_{1,0} ,& \ldots, & \widetilde{\mathcal{H}}_{1,p-1}, & \widetilde{\mathcal{H}}_{2,0}, & \ldots, & \widetilde{\mathcal{H}}_{2,p-1}, & \ldots, & \widetilde{\mathcal{H}}_{\rho,0}, & \ldots ,& \widetilde{\mathcal{H}}_{\rho,q-1} \end{bmatrix} $

and the superscript *T* indicates the transpose of a matrix.

### 2.2. Formulation of fractional model

Temporal memory effects are a common feature of biological processes, and particularly epidemiological dynamics, and they provide important new insights into nonlocal dynamics. Fractional derivatives provide a more efficient way to solve these difficult problems since time-varying kernels are intrinsic to non-integer order derivatives. Fractional derivatives appear in diverse forms in the literature, with the Caputo fractional derivative emerging as the most frequently encountered form. The Caputo operator offers a distinct benefit, as it does not require fractional initial values, unlike classical derivatives. Given these advantageous characteristics, we have opted to employ the Caputo operator in our computational model (1.1).

We add a time-varying kernel in the way described below in order to achieve the power correlation:

$$K(\tau -\theta )=\frac{{\left(\tau -\theta \right)}^{\chi -2}}{\Gamma (\chi -1)}.$$ (2.1)

In integral form, the system (1.1) may be expressed as follows:

2.2 \begin{equation*} 	\begin{aligned} 		\frac{d\mathbb{S}(\tau)}{d\tau} &= \int_{\tau_0}^{\tau} K(\tau - \theta) [\mathcal{B}-\eta\mathbb{S}(\tau)\mathbb{I}(\tau)-\pi\mathbb{S}(\tau)] \ d\theta, \\ 		\frac{d\mathbb{E}(\tau)}{d\tau} &= \int_{\tau_0}^{\tau} K(\tau -\theta) [\eta\mathbb{S}(\tau)\mathbb{I}(\tau)-(\omega+\pi+\Omega)\mathbb{I}(\tau)] \ d\theta, \\ 		\frac{d\mathbb{I}(\tau)}{d\tau} &= \int_{\tau_0}^{\tau} K(\tau - \theta) [\Omega\mathbb{E}(\tau)-(\rho+\pi)\mathbb{I}(\tau)] \ d\theta, \\ 		\frac{d\mathbb{R}(\tau)}{d\tau} &= \int_{\tau_0}^{\tau} K(\tau - \theta) [\rho\mathbb{I}(\tau)+\omega\mathbb{E}(\tau)-\pi\mathbb{R}(\tau)] \ d\theta. 	\end{aligned} \end{equation*}

By inserting the χ−1 order Caputo derivative in (2.2), the following is obtained

$ \begin{aligned} 	^CD^{\chi-1}_\tau \left(\frac{d\mathbb{S}(\tau)}{d\tau}\right) &= ^CD^{\chi-1}_\tau I^{-(\chi-1)}\left(\mathcal{B}-\eta\mathbb{S}(\tau)\mathbb{I}(\tau)-\pi\mathbb{S}(\tau)\right), \\ 	^CD^{\chi-1}_\tau \left(\frac{d\mathbb{E}(\tau)}{d\tau}\right) &=^CD^{\chi-1}_\tau I^{-(\chi-1)}\left(\eta\mathbb{S}(\tau)\mathbb{I}(\tau)-(\omega+\pi+\Omega)\mathbb{I}(\tau)\right), \\ 	^CD^{\chi-1}_\tau \left(\frac{d\mathbb{I}(\tau)}{d\tau}\right) &=^CD^{\chi-1}_\tau I^{-(\chi-1)}\left(\Omega\mathbb{E}(\tau)-(\rho+\pi)\mathbb{I}(\tau)\right), \\ 	^CD^{\chi-1}_\tau \left(\frac{d\mathbb{R}(\tau)}{d\tau}\right) &= ^CD^{\chi-1}_\tau I^{-(\chi-1)}\left(\rho\mathbb{I}(\tau)+\omega\mathbb{E}(\tau)-\pi\mathbb{R}(\tau)\right). \end{aligned} $

Next, The operators $^CD^{\chi-1}_\tau$ and $I^{-(\chi-1)}$ exhibit an interesting property they mutually cancel each other out. Further, to keep the dimension balance on both sides, we re-write the model as follows:

2.3 \begin{equation*} 	\begin{aligned} 		^CD^\chi_\tau \mathbb{S} (\tau) &= \mathcal{B}^\chi-\eta^\chi\mathbb{S}(\tau)\mathbb{I}(\tau)-\pi^\chi\mathbb{S}(\tau), \\ 		^CD^\chi_\tau \mathbb{E} (\tau) &= \eta^\chi\mathbb{S}(\tau)\mathbb{I}(\tau)-(\omega^\chi+\pi^\chi+\Omega^\chi)\mathbb{I}(\tau), \\ 		^CD^\chi_\tau \mathbb{I} (\tau)&= \Omega^\chi\mathbb{E}(\tau)-(\rho^\chi+\pi^\chi)\mathbb{I}(\tau), \\ 		^CD^\chi_\tau \mathbb{R} (\tau) &= \rho^\chi\mathbb{I}(\tau)+\omega^\chi\mathbb{E}(\tau)-\pi^\chi\mathbb{R}(\tau). 	\end{aligned} \end{equation*}

Here, the basic reproductive number for the model (2.3) is given as follows:

$$R_{0}=\frac{\eta^{\chi }\beta^{\chi }}{\pi^{\chi }(\pi^{\chi }+\omega^{\chi }+{Ω}^{\chi })}.$$ (2.4)

The sensitivity index can be computed as follows [19]:

2.5 \begin{eqnarray*} 	\mathcal{S}^{R_0}_{p}=\frac{p}{R_0}\frac{\partial R_0}{\partial p}, \end{eqnarray*}

where *p* represents a parameter of expression (2.4). Using 2.5, we can compute the sensitivity index as follows:

\begin{eqnarray*} 	\mathcal{S}^{R_0}_{\eta}=1>0,\  \mathcal{S}^{R_0}_{\beta}=1>0,\   \mathcal{S}^{R_0}_{\pi}=-1.437<0,\\ 	\mathcal{S}^{R_0}_{\omega}=-0.543<0,\  \mathcal{S}^{R_0}_{\Omega}=-0.0217<0. \end{eqnarray*}

**Figure 2.**
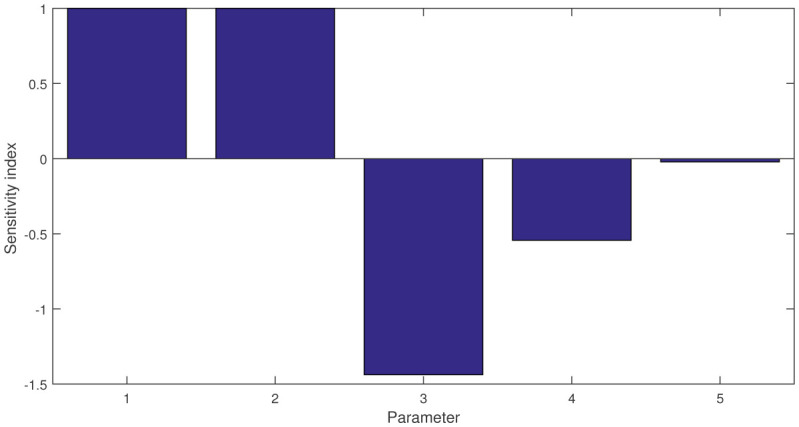
Graphical presentation of sensitivity index

We present the sensitivity index graphically in figure 2 as follows:

## 3. Qualitative analysis

We evaluate the suggested model's well-posedness in this section [43], [44]. To achieve this, we employ methods from fixed-point theory to examine the solutions for the proposed system. The expression on the left-hand side of equation (1.1) assumes the following structure:

3.1 \begin{equation*} 	\begin{aligned} 		\mathbf{X}_1 (\tau, \mathbb{S}, \mathbb{E}, \mathbb{I}, \mathbb{R}) &=\mathcal{B}^\chi-\eta^\chi\mathbb{S}(\tau)\mathbb{I}(\tau)-\pi^\chi\mathbb{S}(\tau), \\ 		\mathbf{X}_2(\tau, \mathbb{S}, \mathbb{E}, \mathbb{I}, \mathbb{R}) &= \eta^\chi\mathbb{S}(\tau)\mathbb{I}(\tau)-(\omega^\chi+\pi^\chi+\Omega^\chi)\mathbb{I}(\tau), \\ 		\mathbf{X}_3(\tau, \mathbb{S}, \mathbb{E}, \mathbb{I}, \mathbb{R}) &= \Omega^\chi\mathbb{E}(\tau)-(\rho^\chi+\pi^\chi)\mathbb{I}(\tau), \\ 		\mathbf{X}_4(\tau, \mathbb{S}, \mathbb{E}, \mathbb{I}, \mathbb{R}) &= \rho^\chi\mathbb{I}(\tau)+\omega^\chi\mathbb{E}(\tau)-\pi^\chi\mathbb{R}(\tau). 	\end{aligned} \end{equation*}

This allows us to systematically assess the model's solution stability and characteristics through well-established mathematical methods. Let the Banach space $\xi = C([0, T] \times \mathcal{R}^4,\mathcal{R})$, and $0 \leq \tau \leq \mathcal{T} < \infty$; then,

$ \|W\|_\xi = \sup_{\tau\in[0,\mathcal{T}]}\left(|\mathbb{S}(\tau)| + |\mathbb{E}(\tau)| + |\mathbb{I}(\tau)| + |\mathbb{R}(\tau)|\right), $

$ W(\tau) = \begin{pmatrix} 	\mathbb{S}(\tau) \\ 	\mathbb{E}(\tau) \\ 	\mathbb{I}(\tau) \\ 	\mathbb{R}(\tau) \end{pmatrix}, \quad W_0 = \begin{pmatrix} 	\mathbb{S}_0 \\ 	\mathbb{E}_0\\ 	\mathbb{I}_0\\ 	\mathbb{R}_0 \\ \end{pmatrix}, \quad \mathcal{X}(\tau,W(\tau)) = \begin{pmatrix} 	\mathbf{X}_1(\tau, \mathbb{S}, \mathbb{E}, \mathbb{I}, \mathbb{R}) \\ 	\mathbf{X}_2(\tau, \mathbb{S}, \mathbb{E}, \mathbb{I}, \mathbb{R}) \\ 	\mathbf{X}_3(\tau, \mathbb{S}, \mathbb{E}, \mathbb{I}, \mathbb{R}) \\ 	\mathbf{X}_4(\tau, \mathbb{S}, \mathbb{E}, \mathbb{I}, \mathbb{R}) \end{pmatrix}(\tau). $

From (3.1), the proposed system (1.1) can takes the following form:

3.2 \begin{equation*} 	^{c}D^\chi W(\tau) = \mathcal{X}(\tau,W(\tau)), \quad \tau \in [0, \mathcal{T}], \end{equation*}

where

$$W(0)=W_{0}.$$

The Caputo initial value problem (3.1) along with definition (2.3) give

3.3 \begin{equation*} 	W(\tau) = W_0 + \int_{0}^{\tau}\frac{(\tau - s)^{\chi-1}}{\Gamma(\chi)}\mathcal{X}(s,W(s))ds, \quad \tau \in [0, \mathcal{T}]. \end{equation*}

We rely on the following assumptions to demonstrate the existence of the (1.1):

**(H1)**: $\exists$ positive constants $Q_{\mathcal{X}}$ and $\Psi_{\mathcal{X}}$ as $\forall$ $M \in \xi$:

$ \|\mathcal{X}(\tau, W(\tau))\| \leq Q_{\mathcal{X}}\|W\| + \Psi_{\mathcal{X}}. $

**(H2)**: $\exists$ a positive constant $\Psi_{\mathcal{X}}$ as $\forall$ $W, W' \in \xi$:

$ \|\mathcal{X}(\tau, W) - \mathcal{X}(\tau, W')\| \leq\chi_{\mathcal{X}}\|W - W'\|. $

**Theorem 3.1.** *[32] Assume that the conditions in* (***H*1**) *hold and $\mathcal{X} : [0, \mathcal{T}] \times \mathcal{R}^4 \rightarrow \mathcal{R}$ is a continous mapping then there is at least one solution for equation (3.3). As a result, at least one solution exists for (1.1) with $vQ_{\mathcal{X}} < 1$, where $v = \frac{\mathcal{T}^\chi}{\Gamma(\chi+1)}$*.

*Proof*. Assuming that (H1) is satisfied, for $\tau \in [0, \mathcal{T}]$, we define:

$ 	L = \{W(\tau) \in \xi : \|W\|_\xi \leq \zeta \}, 	$

as a closed subset of *ξ* with convex properties, where $\zeta \geq \frac{v_0+v \Psi_\mathcal{X}}{1-vQ_\mathcal{X}}$. Further, define

$$T:L\to L,\;\forall \;W\in L,\text{and}|W_{0}|=v_{0},$$

as

$ 	T W(\tau) = W_0 + \frac{1}{\Gamma(\chi)} \int_{0}^{\tau}(\tau - s)^{\chi-1}\mathcal{X}(s,W(s))ds. 	$

Assume that

$ 	\begin{aligned} 		|T W(\tau)|& = \left|W_0 + \frac{1}{\Gamma(\chi)} \int_{0}^{\tau}(\tau - s)^{\chi-1}\mathcal{X}(s,W(s))ds 		\right|, \\&\leq |W_0| + \frac{1}{\Gamma(\chi)} \int_{0}^{\tau}(\tau - s)^{\chi-1}|\mathcal{X}(s,W(s))|ds,\\&  \leq v_0 + vQ_\mathcal{X} \zeta + v\Psi_\mathcal{X},\\ &\leq \zeta. 	\end{aligned} 	$

This implies that $\|T W(\tau)\|_\chi \leq \zeta$; hence, $T(L) \subset L$.

We examine $\tau_{1}<\tau_{2}$ within the interval $[0, \mathcal{T}]$, and we can conclude that *T* exhibits continuity. Our estimation becomes:

$ 	\begin{aligned} 		\|T W(\tau_2) - T W(\tau_1)\| &= \left|\left( 		W_0 + \int^{\tau_2}_{0}\dfrac{(\tau_2 - s)^{\chi-1}}{\Gamma(\chi)}\mathcal{X}(s,W(s))ds\right) - \left(W_0 + \int^{\tau_1}_{0}\frac{(\tau_1 - s)^{\chi-1}}{{\Gamma(\chi)}}\mathcal{X}(s,W(s))ds\right) 		\right|, \\ 		& =\left| \left[\int^{\tau_2}_{0}\dfrac{(\tau_2 - s)^{\chi-1}}{\Gamma(\chi)}-\int^{\tau_1}_{0}\frac{(\tau_1 - s)^{\chi-1}}{{\Gamma(\chi)}}\right]\mathcal{X}(s,W(s))ds\right|, 	\end{aligned} 	$

and so

3.4 \begin{equation*} 		\|T W(\tau_2) - T W(\tau_1)\| \leq \frac{(Q_\mathcal{X}\zeta + v\Psi_\mathcal{X})}{\Gamma(\chi+1)}|\tau_2^{\chi-1} - \tau_1^{\chi-1}|. 	\end{equation*}

Now, the right-hand side of (3.4) approaches 0, as *τ*_2_ approaches *τ*_1_. Therefore, $ \|T W(\tau_2) - T W(\tau_1)\|_\xi \rightarrow 0$; clearly, *T* is uniformly continuous and bounded.

As a result *T* is completely continuous according to the Arzela–Ascoli theorem. Consequently, utilizing Schauder's fixed-point theorem, (1.1) possesses at least one solution.   □

**Theorem 3.2.** *Assuming that (H2) is valid and $\mathcal{T}^\chi \Psi_\mathcal{X} < \Gamma(\chi + 1)$, the measles model (1.1) possesses a singular, unique solution*.

*Proof*. Let *W* and *W*′ be two solutions in *ξ*, and consider T:ξ→ξ as the operator we have

$ 	\begin{aligned} 		\|T W- T W'\|_\xi &= \max_{\tau\in[0,\mathcal{T}]} \left|\int^{\tau}_{0}\dfrac{1}{\Gamma(\chi)}(\tau - s)^{\chi-1}\mathcal{X}(s,W(s))ds-\int^{\tau}_{0}\frac{1}{{\Gamma(\chi)}}(\tau - s)^{\chi-1}\mathcal{X}(s,W(s))ds\right|, \\ 		&\leq \int^{\tau}_{0}\dfrac{(\tau - s)^{\chi-1}}{\Gamma(\chi)}|\mathcal{X}(s,W(s))-\mathcal{X}(s,W'(s))|ds,	 \\ 		&\leq \max_{\tau\in[0,\mathcal{T}]} \int^{\tau}_{0}\dfrac{(\tau - s)^{\chi-1}}{\Gamma(\chi)}\Psi_\mathcal{X}\|W- W'\|_\xi ds, \\ 		&\leq \frac{\mathcal{T}^\chi}{\Gamma(\chi + 1)}\Psi_\mathcal{X}\|W - W'\|_\xi. 	\end{aligned} 	$

The operator *T* exhibits continuity, and consequently, the Banach principle ensures the uniqueness of the solution to (1.1).   □

## 4. Stability criteria

To conduct a comprehensive stability analysis of the proposed model, we revisit several definitions [45]. Consider T:ξ→ξ as a self-map defined by:

TW=W, forW∈ξ. (4.1)

We will say that equation (4.1) exhibits Ulam-Hyers stability if, for every $\mathcal{E} > 0$ and W∈ξ, the solution satisfies:

4.2 \begin{equation*} 	\|\overline{W} - W\|_\xi \leq g_q \mathcal{E}. \end{equation*}

Furthermore, there exists at most one solution *W* of (4.1) with $g_{q}>0$, satisfying

4.3 \begin{equation*} 	\|\overline{W} - W\|_\xi \leq g_q \mathcal{E}. \end{equation*}

**Definition 4.1.** *[?] If, for all $W \in C(\mathbb{R})$ satisfying that W(0)=0 and for every solution W of eq. (4.2), with W being a solution of eq. (4.1), then the following inequality is satisfied:*

$ 	\|\overline{W} - W\|_c \leq W(\mathcal{E}), 	$

*then (4.1) exhibits generalized UHS*.

**Remark 4.2.** *[?] Let $Z(\tau) \in C([0, T];\mathbb{R})$, where $\bar{W}\in \xi$ satisfies (4.3) under the following conditions*

*(i)* $|Z(\tau)| \leq \mathcal{E}$,

*(ii)* $T\bar{W}(\tau )=\bar{W}+Z(\tau )$.

*For our analysis, we examine the perturbed initial value problem denoted in (3.2), i.e.,*

4.4 \begin{equation*} 	^{c}D^\chi_{+0}W(\tau) = W(\tau,W(\tau)) + Z(\tau), \end{equation*}

with the initial condition $W(0)=W_{0}$.

**Lemma 4.3.** *[32]. The below inequality satisfies (4.4):*

$ 	\|W - T W\| \leq a \mathcal{E}, 	$

where

$ 	a = \frac{\mathcal{T}^\chi}{\Gamma(\chi + 1)}. 	$

**Theorem 4.4.** *[46] From Lemma 4.3, the solution to equation (3.2) exhibits UHS when $\frac{T^{\chi }L_{p}}{\Gamma (\chi +1)}<1$, which implies that the solution for the system (1.1) possesses a generalized UHS*.

*Proof*. Consider an arbitrary solution W∈ξ, and let $\bar{W}\in \xi$ be another solution (at most) for (3.2) we have:

$ 	\begin{aligned} 		\|W(\tau) - \overline{W}(\tau)\|_\xi &= \|W(\tau) - T\overline{W}(\tau)\|_\xi \\ 		&\leq \|W(\tau) - T W(\tau)\|_\xi + \|T W(\tau) - T \overline{W}(\tau)\|_\xi \\ 		&\leq a\mathcal{E} + \frac{{\mathcal{T}}^\chi L_p}{\Gamma(\chi+1)} \|W(\tau) - \overline{W}(\tau)\|_\xi, 	\end{aligned} 	$

we conclude that

$ 	\|W - \overline{W}\|_\xi \leq \frac{a\mathcal{E}}{1 - \frac{\mathcal{T}^\chi L_p}{\Gamma(\chi+1)}}. 	$

Demonstrating the UHS of (3.2) also yields the generalized derivation of the UHS.   □

**Definition 4.5.** *Consider (4.1) to demonstrate the stability in the sense of Ulam-Hyers-Rassias for a function $W \in C([0, \mathcal{T}], \mathbb{R})$ for any given $\mathcal{E} > 0$, with $W \in \xi$ as a solution of*

4.5 \begin{equation*} 		\|W - T W\|_\xi \leq W(\tau)\mathcal{E}, \quad \text{for } \tau \in [0, \mathcal{T}], 	\end{equation*}

*there exists a solution* *W* *of (4.1) with* $g_{q}>0$ *that satisfies the below condition*

$ 	\|\overline{W} - W\|_c \leq g_qW(\tau)\mathcal{E}. 	$

**Definition 4.6.** *For $W \in C([0, \mathcal{T}], \mathbb{R})$, assume the existence of a constant $C_{q,W}$. Given that $\mathcal{E} > 0$, consider W as a solution of (4.5) and W as another solution of (4.1); then*,

$ 	\|\overline{W} - W\|_\xi \leq C_{q,W}W(\tau), 	$

*So (4.1) is generalized Ulam–Hyers–Rassias stable*.

**Lemma 4.7.** *The following inequality holds true for (4.3):*

$ 	\|W(\tau) - T W(\tau)\| \leq a \mathcal{E}, 	$

*such that*

$ 	a = \frac{\mathcal{T}^\chi}{\Gamma(\chi + 1)}.$

**Lemma 4.8.** *[?] According to Lemma (4.7), the solution of (4.3) has UHS and generalized UHS whenever $\frac{\mathcal{T}^\chi L_p}{\Gamma(\chi+1)} < 1$*.

*Proof*. Consider an arbitrary solution, W∈ξ, and another solution, $\bar{W}\in \xi$, for (4.3). Here, we have:

$ 	\begin{aligned} 		\|W(\tau) - \overline{W}(\tau)\|_\xi &= \|W(\tau) - T \overline{W}(\tau)\|_\xi, \\ 		&\leq \|W(\tau) - T W(\tau)\|_\chi + \|T W(\tau) - T \overline{W}(\tau)\|_\xi, \\ 		&\leq aW(\tau)\mathcal{E} + \frac{\mathcal{T}^\chi L_p}{\Gamma(\chi+1)} \|W(\tau) - \overline{W}(\tau)\|_\xi. 	\end{aligned} 	$

This gives

$ 	\|W(\tau) - \overline{W}(\tau)\|_\xi \leq \frac{aW(\tau)\mathcal{E}}{1 - \frac{\mathcal{T} ^\chi L_p}{\Gamma(\chi+1)}}. 	$

As a result, (3.2) exhibits UHS, making it a case of generalized UHS    □

## 5. Numerical scheme

Consider that $\mathbb{S}(\tau)$, $\mathbb{E}(\tau)$, $\mathbb{I}(\tau)$, and $\mathbb{R}(\tau)$ belong to $L^{2}[0,1)$, and can be represented by using Haar wavelets series, as follows

$$ \begin{aligned} 	\mathbb{S'}(\tau)=\sum_{j=1}^{\infty}X_jH_j(\tau),\quad& \mathbb{E'}(\tau)=\sum_{j=1}^{\infty}Y_jH_j(\tau), \quad \\ 	\mathbb{I'}(\tau)=\sum_{j=1}^{\infty}Z_jH_j(\tau), \quad& 	\mathbb{R'}(\tau)=\sum_{j=1}^{\infty}W_jH_j(\tau), \end{aligned} $$

where' denotes the derivative, $X_{j},\;Y_{j},\;Z_{j},\;W_{j}$ are Haar series coefficients and $H_{j}(\tau )$ is the discretize Haar function [34]. We integrate the equations governing the transitions of individuals between these compartments to create a model that represents the progression of the epidemic over time. This integration yields a set of equations as follows:

5.1 \begin{equation*} 	\begin{aligned} 		\mathbb{S}(\tau) = \mathbb{S}_0 + \sum_{j=1}^{K} X_jP_{j,1}(\tau), \quad & 		\mathbb{E}(\tau) =  \mathbb{E}_0 + \sum_{j=1}^{K} Y_jP_{j,1}(\tau),\\ 		\mathbb{I}(\tau) = \mathbb{I}_0 + \sum_{j=1}^{K} Z_jP_{j,1}(\tau),\quad& 		\mathbb{R}(\tau) = \mathbb{R}_0 + \sum_{j=1}^{K} W_jP_{j,1}(\tau). 	\end{aligned} \end{equation*}

Here, the operational matrix of integration is denoted by $P_{j,1}(\tau )$ for the Haar wavelet as explained in [34]. Now applying the Caputo derivative, we have

\begin{align*} 	\frac{1}{\Gamma(n - \chi)} \int_{0}^{\tau} \mathbb{S}^{(n)}(\lambda)(\tau - \lambda)^{n - \chi - 1}d\lambda &= \mathcal{B}^\chi-\eta^\chi\mathbb{S}(\tau)\mathbb{I}(\tau)-\pi^\chi\mathbb{S}(\tau), \\ 	\frac{1}{\Gamma(n - \chi)} \int_{0}^{\tau} \mathbb{E}^{(n)}(\lambda)(\tau - \lambda)^{n - \chi - 1}d\lambda &= \eta^\chi\mathbb{S}(\tau)\mathbb{I}(\tau)-(\omega^\chi+\pi^\chi+\Omega^\chi)\mathbb{I}(\tau), \\ 	\frac{1}{\Gamma(n - \chi)} \int_{0}^{\tau} \mathbb{I}^{(n)}(\lambda)(\tau - \lambda)^{n - \chi - 1}d\lambda &= \Omega\mathbb{E}(\tau)-(\rho^\chi+\pi^\chi)\mathbb{I}(\tau), \\ 	\frac{1}{\Gamma(n - \chi)} \int_{0}^{\tau} \mathbb{R}^{(n)}(\lambda)(\tau - \lambda)^{n - \chi - 1}d\lambda &= \rho^\chi\mathbb{I}(\tau)+\omega^\chi\mathbb{E}(\tau)-\pi^\chi\mathbb{R}(\tau). \end{align*}

Assuming $\chi$ to be within the range (0, 1), it follows that *n* = 1, and hence

5.2 \begin{equation*} 	\begin{aligned} 		\frac{1}{\Gamma(1 - \chi)} \int_{0}^{\tau} \mathbb{S'}(\lambda)(\tau - \lambda)^{-\chi }d\lambda &= \mathcal{B}^\chi-\eta^\chi\mathbb{S}(\tau)\mathbb{I}(\tau)-\pi^\chi\mathbb{S}(\tau), \\ 		\frac{1}{\Gamma(1 - \chi)} \int_{0}^{\tau} \mathbb{E'}(\lambda)(\tau - \lambda)^{- \chi }d\lambda &= \eta^\chi\mathbb{S}(\tau)\mathbb{I}(\tau)-(\omega^\chi+\pi^\chi+\Omega^\chi)\mathbb{I}(\tau), \\ 		\frac{1}{\Gamma(1 - \chi)} \int_{0}^{\tau} \mathbb{I'}(\lambda)(\tau - \lambda)^{- \chi }d\lambda &= \Omega^\chi\mathbb{E}(\tau)-(\rho^\chi+\pi^\chi)\mathbb{I}(\tau), \\ 		\frac{1}{\Gamma(1 - \chi)} \int_{0}^{\tau} \mathbb{R'}(\lambda)(\tau - \lambda)^{- \chi }d\lambda &= \rho^\chi\mathbb{I}(\tau)+\omega^\chi\mathbb{E}(\tau)-\pi^\chi\mathbb{R}(\tau). 	\end{aligned} \end{equation*}

Next, from Haar approximations, the above becomes

\begin{align*} 	\frac{1}{\Gamma(1 - \chi)} \int_{0}^{\tau} \sum_{j=1}^{\infty}X_jH_j(\tau)(\lambda)(\tau - \lambda)^{-\chi }d\lambda = &\mathcal{B}^\chi-\eta^\chi\left(\mathbb{S}_0 + \sum_{j=1}^{K}X_jP_{j,1}(\tau)\right)\left(\mathbb{I}_0 + \sum_{j=1}^{K}Z_jP_{j,1}(\tau)\right)\\& - \pi^\chi\left(\mathbb{S}_0 + \sum_{j=1}^{K}X_jP_{j,1}(\tau)\right), \end{align*}

\begin{align*} 	\frac{1}{\Gamma(1 - \chi)} \int_{0}^{\tau} \sum_{j=1}^{\infty}Y_jH_j(\tau)(\lambda)(\tau - \lambda)^{-\chi }d\lambda = &\eta^\chi\left(\mathbb{S}_0 + \sum_{j=1}^{K}X_jP_{j,1}(\tau)\right)\left(\mathbb{I}_0 + \sum_{j=1}^{K}Z_jP_{j,1}(\tau)\right)\\& - (\omega^\chi+\pi^\chi+\Omega^\chi)\left(\mathbb{I}_0 + \sum_{j=1}^{K}Z_jP_{j,1}(\tau)\right), \end{align*}

\begin{align*} 	\frac{1}{\Gamma(1 - \chi)} \int_{0}^{\tau} \sum_{j=1}^{\infty}Z_jH_j(\tau)(\lambda)(\tau - \lambda)^{-\chi }d\lambda = &\Omega^\chi\left(\mathbb{E}_0 + \sum_{j=1}^{K}Y_jP_{j,1}(\tau)\right) - (\rho^\chi+\pi^\chi)\left(\mathbb{I}_0 + \sum_{j=1}^{K}Z_jP_{j,1}(\tau)\right), \end{align*}

\begin{align*} 	\frac{1}{\Gamma(1 - \chi)} \int_{0}^{\tau} \sum_{j=1}^{\infty}W_jH_j(\tau)(\lambda)(\tau - \lambda)^{-\chi }d\lambda = &\rho^\chi\left(\mathbb{I}_0 + \sum_{j=1}^{K}Z_jP_{j,1}(\tau)\right) + \omega^\chi\left(\mathbb{E}_0 + \sum_{j=1}^{K}Y_jP_{j,1}(\tau)\right)\\&-\pi^\chi\left(\mathbb{R}_0 + \sum_{j=1}^{K}W_jP_{j,1}(\tau)\right). \end{align*}

After some calculation we get

\begin{align*} 	&\frac{1}{\Gamma(1 - \chi)} \int_{0}^{\tau} \sum_{j=1}^{\infty}X_jH_j(\tau)(t - \lambda)^{-\chi }d\lambda +\eta^\chi\left(\mathbb{S}_0 + \sum_{j=1}^{K}X_jP_{j,1}(\tau)\right)\left(\mathbb{I}_0 + \sum_{j=1}^{K}Z_jP_{j,1}(\tau)\right)\\&+ \pi^\chi\left(\mathbb{S}_0 + \sum_{j=1}^{K}X_jP_{j,1}(\tau)\right)=0, \end{align*}

\begin{align*} 	&\frac{1}{\Gamma(1 - \chi)} \int_{0}^{\tau} \sum_{j=1}^{\infty}Y_jH_j(\tau)(\tau - \lambda)^{-\chi }d\lambda - \eta^\chi\left(\mathbb{S}_0 + \sum_{j=1}^{K}X_jP_{j,1}(\tau)\right)\left(\mathbb{I}_0 + \sum_{j=1}^{K}Z_jP_{j,1}(\tau)\right)\\& + (\omega^\chi+\pi^\chi+\Omega^\chi)\left(\mathbb{I}_0 + \sum_{j=1}^{K}Z_jP_{j,1}(\tau)\right) = 0, \end{align*}

\begin{align*} 	\frac{1}{\Gamma(1 - \chi)} \int_{0}^{\tau} \sum_{j=1}^{\infty}Z_jH_j(\tau)(\tau - \lambda)^{-\chi }d\lambda - \Omega^\chi\left(\mathbb{E}_0 + \sum_{j=1}^{K}Y_jP_{j,1}(\tau)\right) + (\rho^\chi+\pi^\chi)\left(\mathbb{I}_0 + \sum_{j=1}^{K}Z_jP_{j,1}(\tau)\right) = 0, \end{align*}

\begin{align*} 	&\frac{1}{\Gamma(1 - \chi)} \int_{0}^{\tau} \sum_{j=1}^{\infty}W_jH_j(\tau)(\tau - \lambda)^{-\chi }d\lambda - \rho^\chi\left(\mathbb{I}_0 + \sum_{j=1}^{K}Z_jP_{j,1}(\tau)\right) - \omega^\chi\left(\mathbb{E}_0 + \sum_{j=1}^{K}Y_jP_{j,1}(\tau)\right)\\&+\pi^\chi\left(\mathbb{R}_0 + \sum_{j=1}^{K}W_jP_{j,1}(\tau)\right) = 0. \end{align*}

Here, to approximate the integral in the prior system we have applied Haar's integration formula, as follows [35]:

\begin{align*} 	\int_{x}^{y} f(\tau) \, d\tau &\approx \frac{y - x}{K} \sum_{k=1}^{K} f(\tau_k) = \sum_{k=1}^{K} f\left(x + \frac{(y - x)(k - 0.5)}{K}\right). \end{align*}

Therefore, we obtain

\begin{align*} 	&\frac{\tau}{K\Gamma(1 - \chi)} \sum_{n=1}^{K} \sum_{j=1}^{K}X_jH_j(\lambda_n)(\tau - \lambda_n)^{-\chi } +\eta^\chi\left(\mathbb{S}_0 + \sum_{j=1}^{K}X_jP_{j,1}(\tau)\right)\left(\mathbb{I}_0 + \sum_{j=1}^{K}Z_jP_{j,1}(\tau)\right)\\&+ \pi^\chi\left(\mathbb{S}_0 + \sum_{j=1}^{K}X_jP_{j,1}(\tau)\right)=0, \end{align*}

and

\begin{align*} 	&\frac{\tau}{K\Gamma(1 - \chi)} \sum_{n=1}^{K} \sum_{j=1}^{K}Y_jH_j(\lambda_n)(\tau - \lambda_n)^{-\chi }- \eta^\chi\left(\mathbb{S}_0 + \sum_{j=1}^{K}X_jP_{j,1}(\tau)\right)\left(\mathbb{I}_0 + \sum_{j=1}^{K}Z_jP_{j,1}(\tau)\right)\\& + (\omega^\chi+\pi^\chi+\Omega^\chi)\left(\mathbb{I}_0 + \sum_{j=1}^{K}Z_jP_{j,1}(\tau)\right) = 0, \end{align*}

and

\begin{align*} 	&\frac{\tau}{K\Gamma(1 - \chi)} \sum_{n=1}^{K} \sum_{j=1}^{K}Z_jH_j(\lambda_n)(\tau - \lambda_n)^{-\chi } - \Omega^\chi\left(\mathbb{E}_0 + \sum_{j=1}^{K}Y_jP_{j,1}(\tau)\right) + (\rho^\chi+\pi^\chi)\left(\mathbb{I}_0 + \sum_{j=1}^{K}Z_jP_{j,1}(\tau)\right) = 0, \end{align*}

and

\begin{align*} 	&\frac{\tau}{K\Gamma(1 - \chi)} \sum_{n=1}^{K} \sum_{j=1}^{K}W_jH_j(\lambda_n)(\tau - \lambda_n)^{-\chi } - \rho^\chi\left(\mathbb{I}_0 + \sum_{j=1}^{K}Z_jP_{j,1}(\tau)\right) - \omega^\chi\left(\mathbb{E}_0 + \sum_{j=1}^{K}Y_jP_{j,1}(\tau)\right)\\&+\pi^\chi\left(\mathbb{R}_0 + \sum_{j=1}^{K}W_jP_{j,1}(\tau)\right) = 0. \end{align*}

Now, let

\begin{align*} 	\Theta_{1,i}&=\frac{t}{K\Gamma(1 - \chi)} \sum_{n=1}^{K} \sum_{j=1}^{K}X_jH_j(\lambda_n)(\tau - \lambda_n)^{-\chi } +\eta^\chi\left(\mathbb{S}_0 + \sum_{j=1}^{K}X_jP_{j,1}(\tau)\right)\left(\mathbb{I}_0 + \sum_{j=1}^{K}Z_jP_{j,1}(\tau)\right)\\&+ \pi^\chi\left(\mathbb{S}_0 + \sum_{j=1}^{K}X_jP_{j,1}(\tau)\right), \end{align*}

and

\begin{align*} 	\Theta_{2,i}&=\frac{\tau}{K\Gamma(1 - \chi)} \sum_{n=1}^{K} \sum_{j=1}^{K}Y_jH_j(\lambda_n)(\tau - \lambda_n)^{-\chi }- \eta^\chi\left(\mathbb{S}_0 + \sum_{j=1}^{K}X_jP_{j,1}(\tau)\right)\left(\mathbb{I}_0 + \sum_{j=1}^{K}Z_jP_{j,1}(\tau)\right)\\& + (\omega^\chi+\pi^\chi+\Omega^\chi)\left(\mathbb{I}_0 + \sum_{j=1}^{K}Z_jP_{j,1}(\tau)\right), \end{align*}

and

\begin{align*} 	\Theta_{3,i}&=\frac{\tau}{K\Gamma(1 - \chi)} \sum_{n=1}^{K} \sum_{j=1}^{K}Z_jH_j(\lambda_n)(\tau - \lambda_n)^{-\chi } - \Omega^\chi\left(\mathbb{E}_0 + \sum_{j=1}^{K}Y_jP_{j,1}(\tau)\right) + (\rho^\chi+\pi^\chi)\left(\mathbb{I}_0 + \sum_{j=1}^{K}Z_jP_{j,1}(\tau)\right), \end{align*}

and

\begin{align*} 	\Theta_{4,i}&=\frac{\tau}{K\Gamma(1 - \chi)} \sum_{n=1}^{K} \sum_{j=1}^{K}W_jH_j(\lambda_n)(\tau - \lambda_n)^{-\chi } - \rho^\chi\left(\mathbb{I}_0 + \sum_{j=1}^{K}Z_jP_{j,1}(\tau)\right) - \omega^\chi\left(\mathbb{E}_0 + \sum_{j=1}^{K}Y_jP_{j,1}(\tau)\right)\\&+\pi^\chi\left(\mathbb{R}_0 + \sum_{j=1}^{K}W_jP_{j,1}(\tau)\right). \end{align*}

Combining the nodal points results in this nonlinear system yields

\begin{align*} 	\Theta_{1,i}&=\frac{\tau_i}{K\Gamma(1 - \chi)} \sum_{n=1}^{K} \sum_{j=1}^{K}X_jH_j(\lambda_n)(\tau_i - \lambda_n)^{-\chi } +\eta^\chi\left(\mathbb{S}_0 + \sum_{j=1}^{K}X_jP_{j,1}(\tau_i)\right)\left(\mathbb{I}_0 + \sum_{j=1}^{K}Z_jP_{j,1}(\tau_i)\right)\\&+ \pi^\chi\left(\mathbb{S}_0 + \sum_{j=1}^{K}X_jP_{j,1}(\tau_i)\right), \end{align*}

and

\begin{align*} 	\Theta_{2,i}&=\frac{\tau_i}{K\Gamma(1 - \chi)} \sum_{n=1}^{K} \sum_{j=1}^{K}Y_jH_j(\lambda_n)(\tau_i - \lambda_n)^{-\chi }- \eta^\chi\left(\mathbb{S}_0 + \sum_{j=1}^{K}X_jP_{j,1}(\tau_i)\right)\left(\mathbb{I}_0 + \sum_{j=1}^{K}Z_jP_{j,1}(\tau_i)\right)\\& + (\omega^\chi+\pi^\chi+\Omega^\chi)\left(\mathbb{I}_0 + \sum_{j=1}^{K}Z_jP_{j,1}(\tau_i)\right), \end{align*}

and

\begin{align*} 	\Theta_{3,i}&=\frac{\tau_i}{K\Gamma(1 - \chi)} \sum_{n=1}^{K} \sum_{j=1}^{K}Z_jH_j(\lambda_n)(\tau_i - \lambda_n)^{-\chi } - \Omega^\chi\left(\mathbb{E}_0 + \sum_{j=1}^{K}Y_jP_{j,1}(\tau_i)\right)\\& + (\rho^\chi+\pi^\chi)\left(\mathbb{I}_0 + \sum_{j=1}^{K}Z_jP_{j,1}(\tau_i)\right), \end{align*}

and

\begin{align*} 	\Theta_{4,i}&=\frac{\tau_i}{K\Gamma(1 - \chi)} \sum_{n=1}^{K} \sum_{j=1}^{K}W_jH_j(\lambda_n)(\tau_i - \lambda_n)^{-\chi } - \rho^\chi\left(\mathbb{I}_0 + \sum_{j=1}^{K}Z_jP_{j,1}(\tau_i)\right) - \omega^\chi\left(\mathbb{E}_0 + \sum_{j=1}^{K}Y_jP_{j,1}(\tau_i)\right)\\&+\pi^\chi\left(\mathbb{R}_0 + \sum_{j=1}^{K}W_jP_{j,1}(\tau_i)\right). \end{align*}

Utilizing Broyden's method, we can solve this system. The Jacobian matrix is expressed as follows:

$$J={\left[\begin{array}{c} J_{ip} \end{array}\right]}_{4N\times 4N}\;$$

The Jacobian matrix is determined by evaluating the following partial derivatives:

$$ \begin{aligned} 	\dfrac{\partial \Theta_{1,i}}{\partial X_k}, &\quad \dfrac{\partial \Theta_{1,i}}{\partial Y_k}, &\quad \dfrac{\partial \Theta_{1,i}}{\partial Z_k}, &\quad \dfrac{\partial \Theta_{1,i}}{\partial W_k}, \\ 	\dfrac{\partial \Theta_{2,i}}{\partial X_k}, &\quad \dfrac{\partial \Theta_{2,i}}{\partial Y_k}, &\quad \dfrac{\partial \Theta_{2,i}}{\partial Z_k}, & \quad \dfrac{\partial \Theta_{2,i}}{\partial W_k}, \\ 	\dfrac{\partial \Theta_{3,i}}{\partial X_k}, &\quad \dfrac{\partial \Theta_{3,i}}{\partial Y_k}, &\quad \dfrac{\partial \Theta_{3,i}}{\partial Z_k}, &\quad \dfrac{\partial \Theta_{3,i}}{\partial W_k}, \\ 	\dfrac{\partial \Theta_{4,i}}{\partial X_k}, &\quad \dfrac{\partial \Theta_{4,i}}{\partial Y_k}, &\quad \dfrac{\partial \Theta_{4,i}}{\partial Z_k}, &\quad \dfrac{\partial \Theta_{4,i}}{\partial W_k}. \\ \end{aligned} $$

where

\begin{align*} 	\left\{\begin{array}{l} 		\dfrac{\partial \Theta_{1,i}}{\partial X_k} = \dfrac{\tau_i}{K\Gamma(1 - \chi)} \sum\limits_{n=1}^{K} h_k(\lambda_n)(\tau_i-\lambda_n)^{-\chi} + \eta^\chi \left(\mathbb{I}_0P_{k,1}(\tau_i) + P_{k,1}(\tau_i)\sum\limits_{j=1}^{K}Z_jP_{j,1}(\tau_i) \right) + \pi^\chi P_{k,1}(\tau_i)\\ 		\dfrac{\partial \Theta_{1,i}}{\partial Y_k} = 0\\ 		\dfrac{\partial \Theta_{1,i}}{\partial Z_k} = \eta^\chi \left( \mathbb{S}_0 P_{k,1}(\tau_i) + \sum\limits_{j=1}^{K}X_jP_{j,1}(\tau_i) P_{k,1}(\tau_i)\right)\\ 		\dfrac{\partial \Theta_{1,i}}{\partial W_k} = 0\\ 	\end{array}\right. \end{align*}

and

\begin{align*} 	\left\{\begin{array}{l} 		\dfrac{\partial \Theta_{2,i}}{\partial X_k} = - \eta^\chi \left(\mathbb{I}_0P_{k,1}(\tau_i) + P_{k,1}(\tau_i)\sum\limits_{j=1}^{K}Z_jP_{j,1}(\tau_i) \right) \\		 		\dfrac{\partial \Theta_{2,i}}{\partial Y_k} = \dfrac{\tau_i}{K\Gamma(1 - \chi)} \sum\limits_{n=1}^{K} h_k(\lambda_n)(\tau_i-\lambda_n)^{-\chi} \\	 		\dfrac{\partial \Theta_{2,i}}{\partial Z_k} = -\eta^\chi \left( \mathbb{S}_0 P_{k,1}(\tau_i) + \sum\limits_{j=1}^{K}X_jP_{j,1}(\tau_i) P_{k,1}(\tau_i)\right) + (\omega^\chi+\pi^\chi+\Omega^\chi)P_{k,1}(\tau_i)\\		 		\dfrac{\partial \Theta_{2,i}}{\partial W_k} = 0\\ 	\end{array}\right. \end{align*}

and

\begin{align*} 	\left\{\begin{array}{l} 		\dfrac{\partial \Theta_{3,i}}{\partial X_k} = 0\\		 		\dfrac{\partial \Theta_{3,i}}{\partial Y_k} = -\Omega^\chi P_{k,1}(\tau_i) \\	 		\dfrac{\partial \Theta_{3,i}}{\partial Z_k} = \dfrac{\tau_i}{K\Gamma(1 - \chi)} \sum\limits_{n=1}^{K} h_k(\lambda_n)(\tau_i-\lambda_n)^{-\chi} (\rho^\chi+\pi^\chi)P_{k,1}(\tau_i)\\		 		\dfrac{\partial \Theta_{3,i}}{\partial Z_k} = 0\\ 	\end{array}\right. \end{align*}

and

\begin{align*} 	\left\{\begin{array}{l} 		\dfrac{\partial \Theta_{4,i}}{\partial X_k} = 0\\		 		\dfrac{\partial \Theta_{4,i}}{\partial Y_k} = -\omega^\chi P_{k,1}(\tau_i) \\	 		\dfrac{\partial \Theta_{4,i}}{\partial Z_k} = \rho^\chi P_{k,1}(\tau_i)\\		 		\dfrac{\partial \Theta_{4,i}}{\partial W_k} = \dfrac{t_i}{K\Gamma(1 - \chi)} \sum\limits_{n=1}^{K} h_k(\lambda_n)(\tau_i-\lambda_n)^{-\chi} + \pi^\chi P_{k,1}(\tau_i)\\ 	\end{array}\right. \end{align*}

The unknown coefficients $X_{j},\;Y_{j},\;Z_{j},\;W_{j}$ are obtained from the solution of this above system; also, by substituting these coefficients into (5.1), we obtain the required solutions at nodal positions for $\mathbb{S}(\tau), \ \mathbb{E}(\tau),\ \mathbb{I}(\tau) $ and $\mathbb{R}(\tau)$. The rate of convergence in the experiments, as defined by the formula $r_{\rho }(N)$ [34], [35], were computed by using the subsequent equation:

$ r_\rho(N) = \frac{1}{\log 2} \log \left( \frac{\text{Maximum absolute error at } N^2}{\text{Maximum absolute error at } N} \right). \  $

Next, we will present the graphical outcomes.

### 5.1. Graphical results

In this study, we employed the fractional SEIR model to visualize the dynamics of individuals in different states: susceptible, exposed, infected, and recovered. To ensure the reliability of our investigations, we conducted numerical simulations and graphical analyses for a range of $\chi$ values. To solve this model computationally, we utilized the HWCM. Specifically, we have considered the fractional SEIR model (2.3), where we have set the initial values as follows: $\mathbb{S}(0) = 600$, $\mathbb{E}(0) = 250$, $\mathbb{I}(0) = 100$, and $\mathbb{R}(0) = 50$. It is important to note that we have assumed a constant total population size (N). Additionally, we have defined the following parameter values for the numerical simulations and results: $\mathcal{B} = 0.32, \pi = 0.2, \Omega = 0.01, \rho = 0.2, \eta = 0.01, $ and $ \omega = 0.25$ per day. The resulting figures, encompassing Figures 2 through 5, offer valuable insights into the behaviors of different groups, including susceptible, exposed, infected, and recovered people. Figure 6 displays the fractional-order derivatives of susceptible people for different values of the fractional parameter $\chi$ ranging from 0.75 to 1. It demonstrates a decreasing number of vulnerable people over time as a result of viral exposure, which is consistent with other epidemiological models. In Figure 7, we can observe a clear trend in the population of individuals who have been exposed, showing a consistent and rapid increase as the fractional derivative converges towards its classical solution. The rise can be attributed to an increased proportion of susceptible people being infected and moving into the exposed category. In Figure 8, we can observe an increase in the count of infected individuals as the fractional order approaches 1. This phenomenon is attributed to the heightened sensitivity of the fractional order to different values of the fractional parameter denoted as $\chi$. Figure 9 depicts that as the fractional-order derivative approaches the classical value, we observe a consistent increase in the count of individuals who have successfully recovered, owing mostly to the recovery of infected individuals and thereby playing a significant role in the containment of the transmission of the disease. The population growth in the recovered category will accelerate with an increase in the fractional order. In Figure 10, we visualize the dynamics of the entire SEIR model (2.3) at $\chi=1$. A more accurate representation of the data was made possible by the application of Haar wavelet collocation techniques, which has led to a more exact and trustworthy model. It is also to be emphasized that we have observed a high rate of infected population at the initial stage of infection, but after a certain time, the rate of increase of infected individuals density sloweed down. Further, we have observed that the recovery rate was slow at the initial stage but became high after a certain time. The comparative analysis between the susceptible, exposed, infected, and recovered individuals in the fractional SEIR model has been shown graphically for various values of fractional order. Graphical representations demonstrate how parameters and fractional orders affect the behaviors of susceptible, exposed, infected, and recovered individuals, providing valuable insights into population dynamics during disease outbreaks. This study emphasizes the necessity of employing modern techniques to gain a deeper understanding of the dynamics of the measles disease.

**Figure 3.**
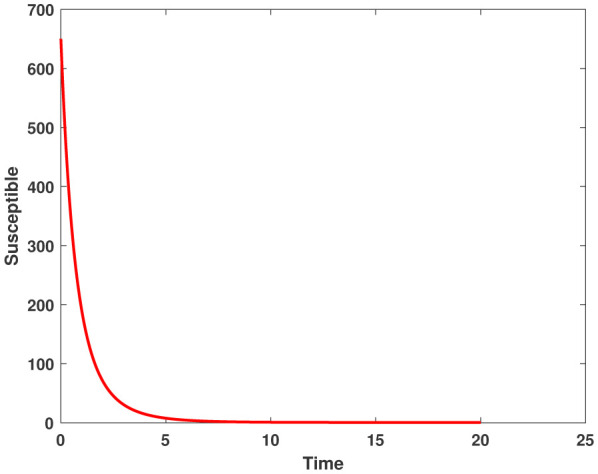
Susceptible population by HWCM at $\chi=1$.

**Figure 4.**
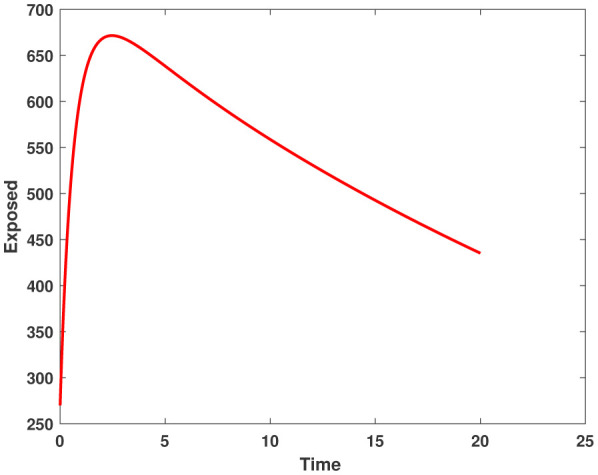
Plot of exposed peoples by HWCM at $\chi=1$.

**Figure 5.**
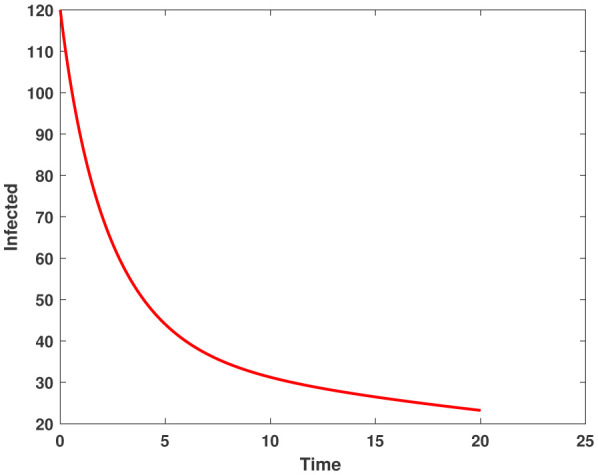
Plot of infected people by HWCM at $\chi=1$.

**Figure 6.**
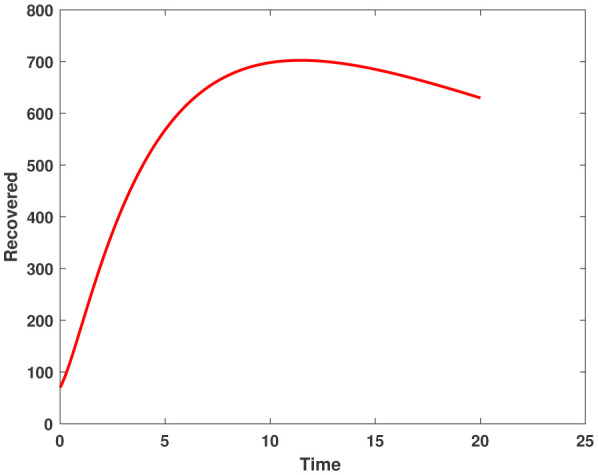
Plot of recovered people by HWCM at $\chi=1$.

**Figure 7.**
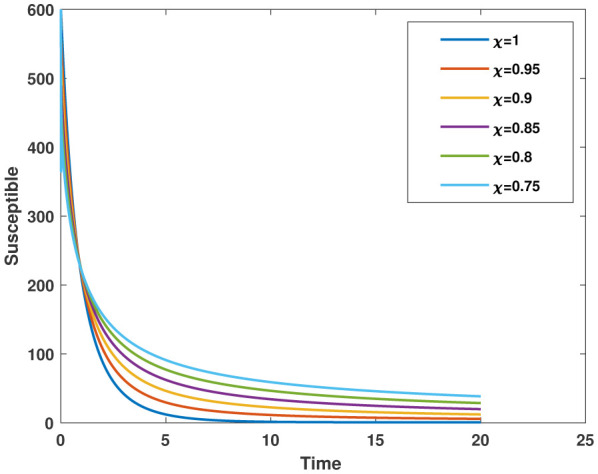
Plot of $\mathbb{S}(\tau)$ for various values of $\chi$.

**Figure 8.**
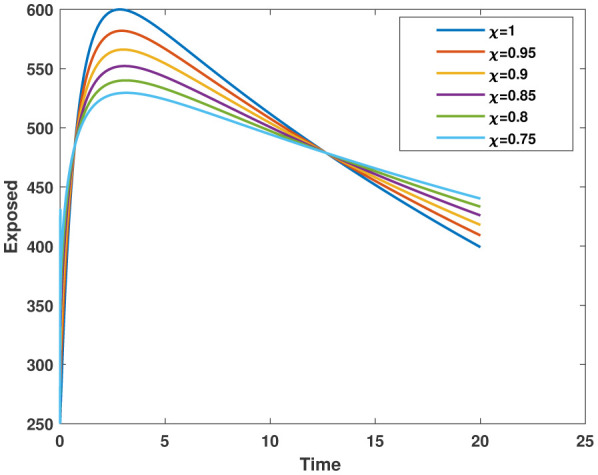
Plot of $\mathbb{E}(\tau)$ for various values of $\chi$.

**Figure 9.**
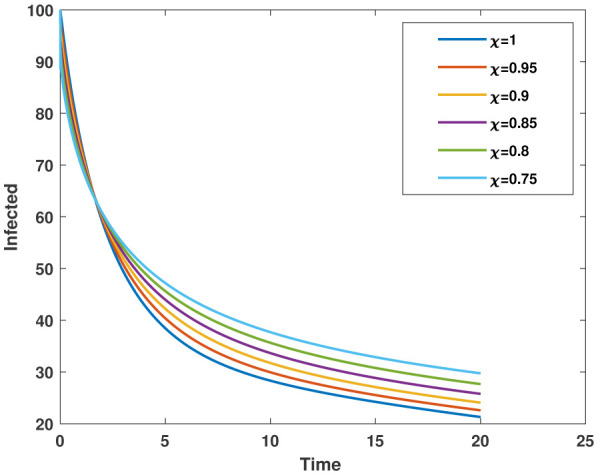
Plot of infected population $\mathbb{I}(\tau)$ for various values of $\chi$.

**Figure 10.**
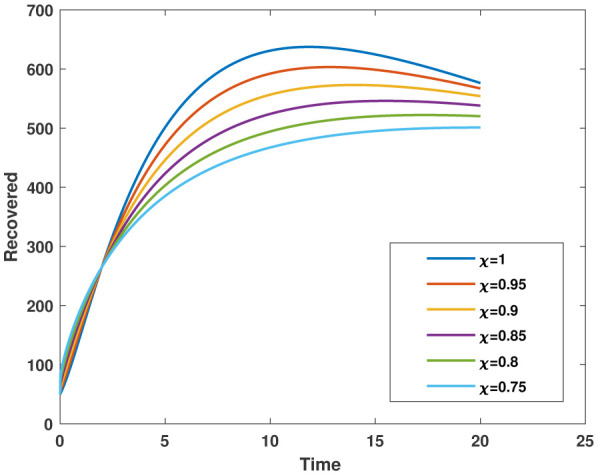
Plot of $\mathbb{R}(\tau)$ for various values of $\chi$.

**Figure 11.**
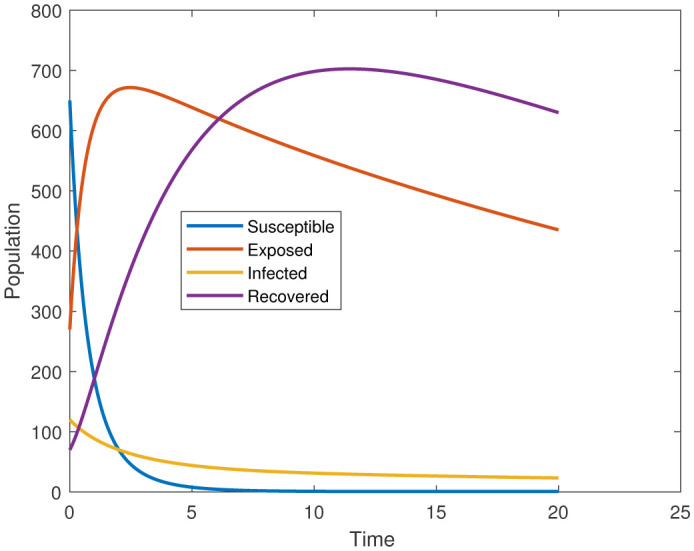
Dynamics of the SEIR system at $\chi=1$.

## 6. Conclusion

We have successfully applied the HWCM to solve fractional measles models efficiently. We investigated the application of fractional derivatives within the Caputo framework to analyze the dynamics of the measles epidemic model. Our method accounts for a number of variables that influence the spread of viruses, and the incorporation of fractional derivatives represents a major advancement in terms of accuracy and effectiveness. To efficiently handle these fractional derivatives, we made use of the Broyden methodology and the HWCM. The Ulam-Hyers method was helpful in determining the stability of the system and offered a vital perspective on the dependability of the model. The major contributions of this study are the application of the HWCM, our investigation of fractional derivatives in the Caputo framework, advancements in accuracy and effectiveness, and our impact on understanding population dynamics. We have shown how the parameters and fractional orders affect the behaviours of susceptible, exposed, infected, and recovered individuals through graphical representations of the dynamics within these compartments across a range of fractional order values. This study emphasizes the need to utilize modern techniques to acquire a greater understanding of the complexities of the measles outbreak. Consequently, the suggested approach is very efficient and applicable to many mathematical models, such as models of cancer treatment, drug targeting systems, and biotherapy. This methodology can be easily implemented in computer programs, and with a small modification to the existing approach, it may be extended to higher degrees. The study's finding suggests that the methodology is not only applicable to measles models but also to various other mathematical models, such as influenza, tuberculosis, or HIV/AIDS, for the analysis and prediction of disease spread dynamics.

## Use of AI tools declaration

The authors declare they have not used Artificial Intelligence (AI) tools in the creation of this article.

## References

[1] Shah K, Khan ZA, Ali A (2020). Haar wavelet collocation approach for the solution of fractional order COVID-19 model using Caputo derivative. Alex Eng J.

[2] Ullah S, Khan MA, Farooq M (2018). A fractional model for the dynamics of TB virus. Chaos Solit Fractals.

[3] Altaf KM, Atangana A (2019). Dynamics of Ebola disease in the framework of different fractional derivatives. Entropy.

[4] Khan H, Alzabut J, Shah A (2023). On fractal-fractional waterborne disease model: A study on theoretical and numerical aspects of solutions via simulations. Fractals.

[5] Zarin R, Ahmed I, Kumam P (2021). Fractional modeling and optimal control analysis of rabies virus under the convex incidence rate. Results Phys.

[6] Khan M, Khan T, Ahmad I (2022). Modeling of Hepatitis B virus transmission with fractional analysis. Math Probl Eng.

[7] Magin R. L. (2012). Fractional calculus in bioengineering: A tool to model complex dynamics. In Proceedings of the 13th International Carpathian Control Conference, High Tatras, Slovakia: IEEE.

[8] Ahmed S, Shah K, Jahan S (2023). An efficient method for the fractional electric circuits based on Fibonacci wavelet. Results Phys.

[9] Caputo M, Mainardi F (1971). A new dissipation model based on memory mechanism. Pure Appl Geophys.

[10] Caputo M, Fabricio M (2015). A new definition of fractional derivative without singular kernel. Prog Fract Differ Appl.

[11] Kilbas AA, Srivastava HM, Trujillo JJ (2006). Theory and Applications of Fractional Differential Equations.

[12] Magin R, Podlubny I (2011). On the fractional signals and systems. Signal Proc.

[13] Rahman MU, Ahmad S, Arfan M (2022). Fractional order mathematical model of serial killing with different choices of control strategy. Fractal Fract.

[14] Shah K, Wenqi L, Raezah AA (2024). Unraveling pine wilt disease: Comparative study of stochastic and deterministic model using spectral method. Expert Syst Appl.

[15] Ahmad Z, Bonanomi G, di Serafino D (2023). Transmission dynamics and sensitivity analysis of pine wilt disease with asymptomatic carriers via fractal-fractional differential operator of Mittag-Leffler kernel. Appl Numer Math.

[16] Malik A, Alkholief M, Aldakheel FM (2022). Sensitivity analysis of COVID-19 with quarantine and vaccination: A fractal-fractional model. Alex Eng J.

[17] Ahmad Z, El-Kafrawy SA, Alandijany TA (2022). A global report on the dynamics of COVID-19 with quarantine and hospitalization: A fractional order model with non-local kernel. Comput Biol Chem.

[18] Sinan M, Ahmad H, Ahmad Z (2022). Fractional mathematical modeling of malaria disease with treatment & insecticides. Results Phys.

[19] Sinan M, Kamal S H A H, Abdeljawad T (2023). Analysis of Nonlinear Mathematical Model of COVID-19 via Fractional-Order Piecewise Derivative. Chaos Theory and Appl.

[20] Aghdaoui H, Alaoui AL, Nisar KS (2021). On analysis and optimal control of a SEIRI epidemic model with general incidence rate. Results Phys.

[21] Hadi F, Amin R, Khan I (2023). Numerical solutions of nonlinear delay integro-differential equations using Haar wavelet collocation method. Fractals.

[22] Amin R, Hafsa, Hadi F (2023). Solution of Variable-Order Nonlinear Fractional Differential Equations Using Haar Wavelet Collocation Technique. Fractals.

[23] Scindia PS, Nisar KS (2023). Ulam's type stability of impulsive delay integrodifferential equations in Banach spaces. Int J Nonlin Sci Num.

[24] Berhe HW, Makinde OD (2020). Computational modelling and optimal control of measles epidemic in human population. Biosyst.

[25] Berhe HW, Makinde OD, Theuri DM (2019). Co-dynamics of measles and dysentery diarrhea diseases with optimal control and cost-effectiveness analysis. Appl Math Comput.

[26] Yano TK, Makinde OD, Malonza DM (2016). Modelling childhood disease outbreak in a community with inflow of susceptible and vaccinated new-born. Global J Pure Appl Math.

[27] Yang Y, Xu L (2019). Stability of a fractional order SEIR model with general incidence. Appl Math Lett.

[28] Hashim I, Abdulaziz O, Momani S (2009). Homotopy analysis method for fractional IVPs. Commun Nonlinear Sci Numer Simul.

[29] Kumar M (2022). Spreading behavior of biological SIR system of a COVID-19 disease through a fast Taylor wavelet based numerical algorithm. Results Control Optim.

[30] Sazmand A, Behroozifar M (2018). Application Jacobi spectral method for solving the time-fractional differential equation. J Comput Appl Math.

[31] Walnut DF (2013). An introduction to wavelet analysis.

[32] Raizah Z, Zarin R (2023). Advancing COVID-19 understanding: Simulating omicron variant spread using fractional-order models and haar wavelet collocation. Math.

[33] Ahmed S, Jahan S, Nisar KS (2023). Hybrid fibonacci wavelet method to solve fractional-order logistic growth model. Math Methods Appl Sci.

[34] Lepik Ü (2009). Solving fractional integral equations by the Haar wavelet method. Appl Math Comput.

[35] Li Y, Zhao W (2010). Haar wavelet operational matrix of fractional order integration and its applications in solving the fractional order differential equations. Appl Math Comput.

[36] Kumar S, Kumar R, Momani S (2023). A study on fractional COVID-19 disease model by using Hermite wavelets. Math Methods Appl Sci.

[37] Kumar S, Ahmadian A, Kumar R (2022). An efficient numerical method for fractional SIR epidemic model of infectious disease by using Bernstein wavelets. Math.

[38] Amin R, Yúzbaşı S, Nazir S (2022). Efficient Numerical Scheme for the Solution of HIV Infection CD4+ T-Cells Using Haar Wavelet Technique. CMES-Comput Model Eng Sci.

[39] Prakash B, Setia A, Alapatt D (2017). Numerical solution of nonlinear fractional SEIR epidemic model by using Haar wavelets. J Comput Sci.

[40] Momoh AA, Ibrahim MO, Uwanta IJ (2013). Mathematical model for control of measles epidemiology. Int J Pure Appl Math.

[41] Kumar S, Kumar R, Osman MS (2021). A wavelet based numerical scheme for fractional order SEIR epidemic of measles by using Genocchi polynomials. Numer Methods Partial Differ Equ.

[42] Al-Smadi MH, Gumah GN (2014). On the homotopy analysis method for fractional SEIR epidemic model. J Appl Sci Eng Tech.

[43] Zeidler E (2013). Nonlinear Functional Analysis and Its Applications, IV: Applications to Mathematical Physics.

[44] Khan A, Khan H, Gómez-Aguilar JF (2019). Existence and Hyers-Ulam stability for a nonlinear singular fractional differential equations with Mittag-Leffler kernel. Chaos Solit Fractals.

[45] Jamal N, Sarwar M, Khashan MM (2021). Hyers-Ulam stability and existence criteria for the solution of second-order fuzzy differential equations. Journal of Function Spaces.

[46] Khan H, Chen W, Khan A (2018). Hyers–Ulam stability and existence criteria for coupled fractional differential equations involving p-Laplacian operator. Adv Differ Equ.

